# Supplementary material for: Reconciling Mining with the Conservation of Cave Biodiversity: A Quantitative Baseline to Help Establish Conservation Priorities
Source: PLoS One. 2016 Dec 20;11(12):e0168348. doi: 10.1371/journal.pone.0168348 (PMC5173368; doi:10.1371/journal.pone.0168348)
Supplement: S1 Dataset — (ZIP) [file pone.0168348.s002.zip › Taxa/Serra Norte/SN_2013/N4WS/Lista N4WS-02.pdf]

CAVIDADE N4WS-0002

Classe	Ordem	Fam/Outros	Gên/Outros	Espécie	S-U	U-S	Zon.
Arachnida	Acari	Argasidae	*Ornithodoros*	sp.1	X	X	P
Arachnida	Amblypygi	Phrynidae	*Heterophrynus*	*longicornis*	1	1	P
Arachnida	Araneae	Filistatidae		jovem		X	E
Arachnida	Araneae	Ochyroceratidae	*Speocera*	sp.1	X	X	P
Arachnida	Araneae	Pholcidae	*Mesabolivar*	*eberhard*	X		E
Arachnida	Araneae	Scytodidae	*Scytodes*	*globula*	3		E/P
Arachnida	Araneae	Theridiidae	*Achaearanea*	sp.1		X	E
Arachnida	Araneae	Theridiosomatidae	*Plato*	sp.1	X		E/P
Arachnida	Araneae	Uloboridae		jovem	X		E
Arachnida	Pseudoscorpiones	Chernetidae	*Spelaeochernes*	sp.1		X	E/P
Entognatha	Collembola	Paronellidae		sp.4	X	X	P
Insecta	Coleoptera	Tenebrionidae		jovem		X	P
Insecta	Diptera	Cecidomyiidae	Cecidomyiinae	sp.		X	P
Insecta	Diptera	Chironomidae		sp.		X	E
Insecta	Heteroptera	Reduviidae	Reduviinae	jovem		2	E
Insecta	Hymenoptera	Formicidae	*Nylanderia*	sp.2	X		E
Insecta	Hymenoptera	Formicidae	*Odontomachus*	sp.1	X		E
Insecta	Hymenoptera	Formicidae	*Solenopsis*	sp.1	X		P
Insecta	Hymenoptera	Formicidae	*Solenopsis*	sp.2		X	E
Insecta	Lepidoptera	Noctuoidea		sp.1	1		E
Insecta	Neuroptera	Mantispidae	*Plega*	sp.	1		P
Insecta	Neuroptera	Myrmeleontidae		jovem	48	52	E
Insecta	Orthoptera	Phalangopsidae	*Phalangopsis*	sp.	8	12	P
Insecta	Psocoptera	Epipsocidae	*Hinduipsocus*	sp.1		X	E
Insecta	Psocoptera	Psyllipsocidae	*Psocathropos*	sp.1		X	E
Amphibia	Anura	Strabomantidae	*Pristimantis*	cf. *fenestratus*	1		P
Mammalia	Chiroptera	Emballonuridae	*Peropteryx*	sp.	4	4	P
